# Glucocorticoids and checkpoint tyrosine kinase inhibitors stimulate rat pancreatic beta cell proliferation differentially

**DOI:** 10.1371/journal.pone.0212210

**Published:** 2019-02-19

**Authors:** Sarah Akbib, Jordy Stichelmans, Geert Stangé, Zhidong Ling, Zerihun Assefa, Karine H. Hellemans

**Affiliations:** 1 Unit Diabetes Pathology and Therapy, Diabetes Research Cluster, Vrije Universiteit Brussel, Brussels, Belgium; 2 Beta Cell Bank, University Hospital Brussels, Brussels, Belgium; 3 Center for Beta Cell Therapy in Diabetes, Brussels, Belgium; Broad Institute, UNITED STATES

## Abstract

Cell therapy for diabetes could benefit from the identification of small-molecule compounds that increase the number of functional pancreatic beta cells. Using a newly developed screening assay, we previously identified glucocorticoids as potent stimulators of human and rat beta cell proliferation. We now compare the stimulatory action of these steroid hormones to a selection of checkpoint tyrosine kinase inhibitors that were also found to activate the cell cycle-in beta cells and analyzed their respective effects on DNA-synthesis, beta cell numbers and expression of cell cycle regulators. Our data using glucocorticoids in combination with a receptor antagonist, mifepristone, show that 48h exposure is sufficient to allow beta cells to pass the cell cycle restriction point and to become committed to cell division regardless of sustained glucocorticoid-signaling. To reach the end-point of mitosis another 40h is required. Within 14 days glucocorticoids stimulate up to 75% of the cells to undergo mitosis, which indicates that these steroid hormones act as proliferation competence-inducing factors. In contrast, by correlating thymidine-analogue incorporation to changes in absolute cell numbers, we show that the checkpoint kinase inhibitors, as compared to glucocorticoids, stimulate DNA-synthesis only during a short time-window in a minority of cells, insufficient to give a measurable increase of beta cell numbers. Glucocorticoids, but not the kinase inhibitors, were also found to induce changes in the expression of checkpoint regulators. Our data, using checkpoint kinase-specific inhibitors further point to a role for Chk1 and Cdk1 in G1/S transition and progression of beta cells through the cell cycle upon stimulation with glucocorticoids.

## Introduction

Beta cell replacement therapy and regeneration of the endogenous beta cell mass are both considered to be hopeful approaches to cure type 1 diabetic patients [1–3]. However, the shortage in human donor organs, the low yield that characterizes islet isolations and the absence of drugs with robust mitogenic effects on beta cells, or efficient protocols to differentiate stem cells to functional mature beta cells hamper progression. The use of cell replacement or cell regeneration therapy as a first-line therapy for type 1 diabetes thus depends on the development of conditions that would allow for the generation of new, or expansion of existing beta cells, both *in vitro* or *in vivo* [1–3].

In this context several drug-screening platforms have been developed and multiple stimulatory compounds have been described over the last decade [4–7]. Thus far however, these efforts did not lead to the development of compounds suitable to expand functional beta cells. Most screening approaches focus on stimulation of DNA-synthesis as a read-out, but fail to identify compounds that induce a noticeable beta cell expansion. Therefore, we previously validated a high-content screening assay in which acute stimulation of DNA-synthesis is coupled to measuring changes in absolute beta cell numbers after prolonged incubation [8]. Using this strategy, we identified glucocorticoids (GCs) as the most potent stimulators of rat and human beta cell proliferation [9]. Sustained incubation with these steroidal hormones, acting via the glucocorticoid receptor, resulted in a near doubling of beta cell numbers within fourteen days. The stimulatory effect was limited to a subpopulation of metabolically active adult beta cells, whereas GCs were toxic for immature cells. Moreover, GC-expanded beta cells were able to restore glycaemia when transplanted in diabetic mice [9]. Of interest, GCs were recently also identified as stimulators of beta-cell replication and regeneration in a zebra fish model [10].

In the current study, we compare the effect of these hormones on cell cycle regulation, to another potent family of proliferation-stimulatory compounds, namely tyrosine kinase inhibitors (TKIs). Although TKIs are well known for their ability to change the activation status of cell cycle regulators [11–13], a stimulatory effect on beta cell proliferation has not been reported before. Further characterization of these compounds as inducers of beta cell replication is of interest as recent studies with TKIs to treat a variety of cancers have indicated antihyperglycemic properties [14,15]. Their potential for the treatment of diabetes is under evaluation.

The results presented in this manuscript concentrate on the differences between GC and TKIs. GCs seem to act as replication competence-inducing agents that stimulate mature beta cells to pass the restriction point and continue to recruit new cells into the cell cycle throughout culture. In contrast, we could not demonstrate an increase in beta cell numbers with TKIs that solely seem to act as cell cycle progression factors that allow beta cells to enter the next phase of the cell cycle without really recruiting the cells into a proliferative activity. Moreover, our data using specific checkpoint kinase inhibitors also point to a central role for Chk1 and Cdk1 in beta cell proliferation.

## Materials and methods

### Chemicals

The Library of Pharmacologically-Active Compounds (LOPAC), 6α-methylprednisolone (6MP), dexamethasone (Dex), mifepristone, PD166285 (PD16), PD173952 (PD17), NU6027 and CGP7454A were purchased from Sigma-Aldrich; dasatinib, MK-1775 and CHIR-124 from SelleckChem. All compounds were diluted in DMSO to 10 mM stock solutions and kept at -20°C.

### Isolation and culture of adult rat beta cells

Isolation protocols were approved by the Ethical Committee for Animal Experiments of the Vrije Universiteit Brussel and conducted according to the European Community Council Directive (86/609/EEC). Beta cells from 8–10 week-old male Wistar rats (Janvier Bioservices) were purified by FACS on the basis of their NAD(P)H-autofluorescence to a mean purity of 92 ± 3% insulin-positive cells with 5 ± 3% glucagon positive cells, as described before [16–18]; seeded on 804G-matrix coated 384-well or 6-well plates (Greiner Bio-one) and cultured for a maximum of 15 days in serum-free Ham’s F10 medium (Thermo Fisher Scientific) at 10mM glucose. Culture media were supplemented with 50μM 3-isobutyl-1-methylxanthine (IBMX), 2mM L-glutamine, 0.5% Albumax I, penicillin (0.075mg/ml), streptomycin (0.1mg/ml) and refreshed every 72h [19]. These culture conditions maintain beta cell differentiation and viability over a prolonged period [19]. The 804G-cell line was a gift of Dr T. Otonkoski (University of Helsinki, Finland).

### Isolation, purification and culture of human beta cells

Human donor endocrine-enriched cell clusters (4 donors, 21–65 yr of age) were obtained from the UZBrussels Beta Cell Bank according to Eurotransplant criteria for their use in clinical transplantation and associated projects. Experimental protocols were approved by the UZBrussels institutional ethical committee (CME 2005/118, 2010/193, 2017/043). After 1–3 weeks of serum-free culture [20], the cell clusters were enzymatically dispersed and incubated with the zinc-binding fluorochrome 6-methoxy-(8-p-toluenesulfonamido)quinolone (Thermo Fisher Scientific) and FACS purified. The beta cell-enriched populations were reaggregated overnight and seeded on collagen type IV (Sigma-Aldrich) coated 384-well plates and cultured in serum-free Ham’s F10 medium, supplemented with 7.5mM glucose, and 0.5% human serum albumin (C.A.F.-D.C.F., Red Cross, Belgium).

### Analysis of beta cell viability, cell number and DNA-synthesis

All procedures for the analysis of absolute cell numbers, viability and proliferation activity have been detailed before [8,9]. In short, freshly isolated cells were seeded and incubated overnight before test compounds (or vehicle) were added for 5 to 14 days. The absolute number and percent living or dead cells was determined by Hoechst-Propidium iodide (Ho-PI) staining on day 1 (24h after seeding) and followed over 14 days using a Pathway Bioimager (BD Biosciences). Whole-wells were imaged and analyzed using IPLab® and Attovision® software. The absolute number of living cells (PI-negative) was expressed relative to the number of cells in the control condition (fold change), or as a percent of day 1 numbers. The number of cells involved in DNA-synthesis was demonstrated by incubating the cells the last 72h of culture with 10 μM 5-ethynyl-2-deoxyuridine (EdU). EdU-positive cells were visualized using the Click-iT EdU Alexa-Fluor Imaging Kit (Thermo-Fisher Scientific) and expressed as percentage of the total number of cells, or relative to the indicated control condition (Fold Change).

### Quantitative PCR

RNA was extracted using RNeasy columns (Qiagen) and converted to cDNA using the High-capacity cDNA archive Kit (Applied Biosystems). qPCR was performed using Taqman Universal PCR Master Mix and MGB probes (Applied Biosystems) and amplified using an ABI Prism 7700 System. The expression levels of target genes were normalized to four housekeeping genes: β-actin (NM_03114), HPRT1 (Rn01527840_m1), rplp2 (Rn01479927_g1) and psmc5 (Rn00579821_m1). Differences between samples were analyzed using the comparative Ct method (ΔΔCt). Gene specific arrays were obtained from Thermo-Fisher Scientific. Details are available on request.

### Immunocytochemistry

For immunocytochemistry cells were fixed on the indicated days with 4% formaldehyde, incubated overnight with primary antibodies (guinea pig anti-insulin or rabbit anti-glucagon, gift from C. Van Schravendijk) and visualized with Alexa-fluor secondary antibodies (Jackson ImmunoResearch, Suffolk, UK) counterstained with 4',6-diamidino-2-phenylindole (DAPI; Sigma-Aldrich, St Louis, MO, USA).

### Statistical analysis

Data are presented as mean ± standard deviation or standard error of the mean of n independent experiments. Statistical significance of differences was assessed using GraphPad prism 6.01 (GraphPad Software, San Diego, USA). Results from library screening were analyzed by calculating the standard score (z-score) [21].

## Results

### High-content screening identifies checkpoint kinase inhibitors as potent activators of DNA-synthesis in rat and human beta cells

A library of 1,280 pharmacological active compounds was screened for compounds that recruit adult rat beta cells into proliferative activity during a 6-day culture period. Test compounds (1μM) were added for 5-days to serum-free HamF10 medium supplemented with IBMX (50μM), EdU (10μM) was added the last 72h of culture. The z-score was calculated from the percentage of EdU-positive cells. This screening had previously identified glucocorticoids as robust inducers of DNA-synthesis, with an average z-score of 4.7 ± 0.8 [9]. We now compare GCs to the second most powerful inducers of DNA synthesis identified during this screening, i.e. tyrosine kinase inhibitors. The kinase/phosphatase class of the LOPAC library contains in total 15 compounds, of which 5 tyrosine kinase inhibitors (further denoted as TKIs) showed a significant effect on DNA synthesis, with an average z-score of 3.2 ± 0.7 (**Table 1**). None of these compounds showed a negative impact on cell viability. Their stimulatory effect was found specific for beta cells as no EdU-labeling was observed in contaminating alpha cells (**S1 Fig**).

**Table 1:** TKIs stimulate DNA-synthesis in adult rat beta cells.

Name	Day 15% living cells	z-score	Description
PD173952	96	4.3	Src family, Wee1 kinase inhibitor.
PD161570	98	3.7	Src family, FGF-1 receptor kinase inhibitor
PD166285 hydrate	97	3.3	Src family, Wee1, Myt1 and Chk1 kinase inhibitor
PD169316	98	3.1	p38 MAP kinase inhibitor
PD407824	98	2.5	Wee1, Chk1 kinase inhibitor
IRAK-1/4 Inhibitor I	99	2.5	IL-1 receptor-associated kinase 1/4 inhibitor

Purified rat beta cells were pre-incubated overnight in control medium followed by 5-day culture with test compounds at 1μM. The last 72h, EdU was added to label proliferating cells. The percentage living cells was determined as the number of propidium iodide-negative cells compared to the total number of Hoechst-positive cells. The percentage EdU-positive cells was determined on day 6 and used to determine the z-score (n = 1).

**Fig 1:**
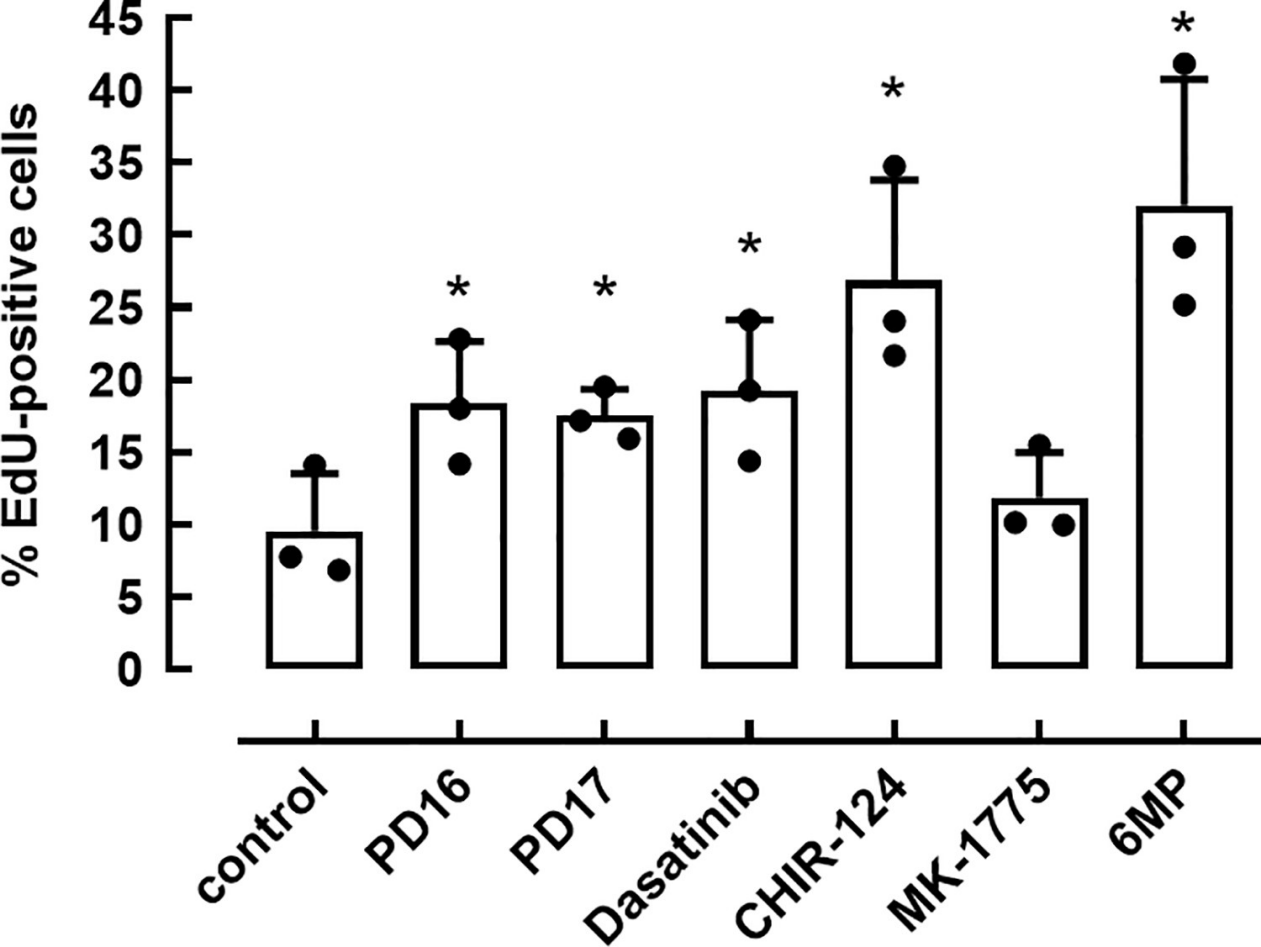
Effect of checkpoint kinase inhibitors on DNA-synthesis. Purified rat beta cells were cultured with 6MP and indicated inhibitors PD16, PD17, dasatinib (Src inhibitor), CHIR-124 (Chk1 inhibitor) or MK-1775 (Wee1 inhibitor). EdU was added between day 3–6 and the percent of EdU-positive cells were measured on day 6. Data represent mean ± SD (n = 3), black dots represent actual data points. Statistical differences were analyzed by Two-way ANOVA with Tukey’s correction for multiple comparisons. * p<0.05, as compared to control cells.

TKIs have been reported to abrogate the G1/S checkpoint that prevents cell cycle progression in absence of an adequate signal by inhibiting several checkpoint kinases and Src-family kinases, which results in the activation of Cdk:cyclin complexes [11–13]. From the 5 identified TKIs we selected PD166285 (PD16) and PD173952 (PD17) for further experiments. However, since PD16 and PD17 can inhibit various checkpoint kinases (Chk1, Wee1 and Myt1), as well as Src-family kinases (such as Lyn and Fyn), and other kinases (**Table 1**), we examined whether their stimulatory effect on DNA synthesis could be reproduced using more specific checkpoint kinase inhibitors: MK-1775 (a Wee1-inhibitor, 1μM), dasatinib (a Src-family kinase inhibitor, 1μM) and CHIR-124 (a Chk1-inhibitor, 0.1μM). Rat beta cells were exposed to these inhibitors as described above. MK-1775 showed no stimulatory effect, but dasatinib stimulated DNA-synthesis to levels comparable to those measured with PD16 and PD17, and CHIR-124 even up to the level of stimulation induced by 6-methylprednisolone (6MP), a synthetic GC (**Fig 1**) [9].

PD16, PD17 and CHIR-124 were also found to stimulate DNA-synthesis in human beta cells comparable to the level of stimulation observed for 6MP (**Table 2**). The TKIs increased the percent of EdU-positive human beta cells in all tested preparations with a wide variation in individual responses to 0.3–4.2% above the 0.0–0.5% present in control cells. In view of the limited availability of human donor beta cells, all subsequent experiments were performed with purified adult rat beta cells.

**Table 2:** GCs and TKIs induce DNA-synthesis in human beta cells.

**Donor Charecteristics**		**Percentage Insulin/EdU double positive beta cells**				
**Age, yr**	**seks**	**Control**	**6MP**	**PD16**	**PD17**	**CHIR-124**
21	Male	0.0 ± 0.0	0.3 ± 0.1	3.9 ± 0.5	4.2 ± 0.5	2.6 ± 0.3
50	Female	0.5 ± 0.1	2.5 ± 0.1	1.1 ± 0.4	0.7 ± 0.4	2.4 ± 0.3
56	Female	0.0 ± 0.1	0.1 ± 0.1	0.3 ± 0.2	0.4 ± 0.3	0.5 ± 0.2
65	Female	0.1 ± 0.0	0.4 ± 0.2	0.8 ± 0.2	0.6 ± 0.1	0.8 ± 0.2
Average ± SD		0.2 ± 0.1	0.9 ± 0.6*	1.5 ± 0.8	1.4 ± 0.9*	1.6 ± 0.5*
**Donor Charecteristics**		**Percentage Insulin/EdU double positive beta cells**				
**Age, yr**	**seks**	**Control**	**6MP**	**PD16**	**PD17**	**CHIR-124**
21	Male	0.0 ± 0.0	0.3 ± 0.1	3.9 ± 0.5	4.2 ± 0.5	2.6 ± 0.3
50	Female	0.5 ± 0.1	2.5 ± 0.1	1.1 ± 0.4	0.7 ± 0.4	2.4 ± 0.3
56	Female	0.0 ± 0.1	0.1 ± 0.1	0.3 ± 0.2	0.4 ± 0.3	0.5 ± 0.2
65	Female	0.1 ± 0.0	0.4 ± 0.2	0.8 ± 0.2	0.6 ± 0.1	0.8 ± 0.2
Average ± SD		0.2 ± 0.1	0.9 ± 0.6*	1.5 ± 0.8	1.4 ± 0.9*	1.6 ± 0.5*

Beta cell-enriched populations of 4 different donors were cultured for 6 days in 7.5 mM glucose without or with indicated compounds at 1μM. EdU was added for the last 72h. The percentage of EdU-positive cells was determined in the insulin-positive population. For each donor preparation the average and SD of triplicate measurements are shown per condition. The data were analyzed with Two-way repeated measures ANOVA with Dunnett’s correction

* p<0.001 compared to cells under control conditions.

### Cdk1 plays a central role in cell cycle progression induced by checkpoint kinase inhibitors and glucocorticoids

**Fig 2:**
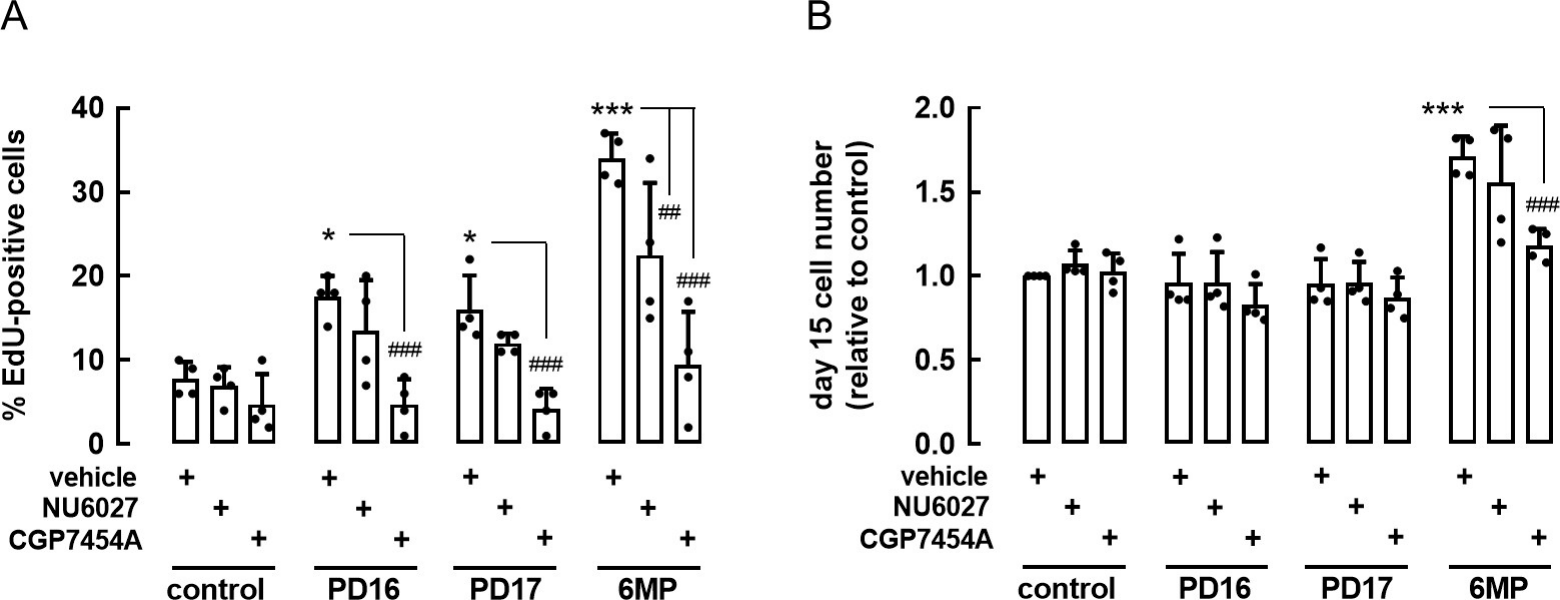
Effect of Cdk1 and Cdk2 inhibition on DNA-synthesis and cell numbers in cells exposed to 6MP or TKIs. Purified rat beta cells were cultured with 6MP and indicated inhibitors CGP7454A (Cdk1 inhibitor, 1μM) and NU6027 (Cdk2 inhibitor, 1μM). Shown are effects of compounds on percentage EdU^+^ cells (A) and day 15 cell numbers (B). Data represent mean ± SD (n = 4), black dots represent the actual data points. Statistical differences were analyzed by Two-way ANOVA with Tukey’s correction for multiple comparisons. * p<0.05, ** p<0.01, *** p<0.001, as compared to control cells. ### p<0.001 as compared to 6MP.

As inhibition of Chk1 may activate various Cdk:cyclin complexes [11–13,22], we evaluated the effect of specific Cdk-inhibitors (NU6027, a Cdk2-inhibitor and CGP-74514A, a Cdk1-inhibitor) on PD16, PD17 and 6MP-induced proliferation. NU6027 (1μM) and CGP-74514A (1μM) did not affect basal EdU incorporation (**Fig 2A**) nor cell viability (not shown), but in combination with TKIs DNA-synthesis was suppressed by 75% by the Cdk1-inhibitor, whereas no significant effect was observed with the Cdk2-inhibitor. These results suggest that TKIs remove the G1/S checkpoint in a subset of beta cells mainly by alleviating the inhibitory phosphorylation of Cdk1 and to a lesser extent Cdk2. The same observation was made in 6MP-activated beta cells. Inhibition of Cdk2 resulted in a modest reduction (35%) of the percent EdU-positive cells, whereas Cdk1 inhibition showed a 60% reduction in the number of EdU-positive cells (**Fig 2A**). The difference between Cdk1 and Cdk2 was also reflected in the number of cells measured after 15 days. Inhibition of Cdk1 completely blocked 6MP-induced beta cell expansion, while inhibition of Cdk2 showed no effect on cell numbers (**Fig 2B**). To our surprise, and despite their clear stimulatory effect on DNA-synthesis, cell numbers remained constant in the presence of PD16 or PD17. Consequently we also found no effect of NU6027 and CGP-74514A on cell numbers when combined with TKI’s.

The results shown in **Figs 1** and **2** suggest a role of Chk1 and Cdk1 in beta cell proliferation. To evaluate this further, we examined the effect of GCs and TKIs on the mRNA levels of different cell cycle regulators. RNA was extracted after 6 days culture and used for qPCR analysis (**Table 3**). No significant change in transcript levels was observed for cells treated with TKIs; whereas 6MP-treated cells showed significantly increased transcript levels for regulators of the G1/S checkpoint: Cdk1 (5-fold) and cyclin E (3-fold), as well as Myt1 (3-fold) and Chk1 (4-fold, although not statistically significant due to large interexperimental variation). In addition, 6MP-treatment leds to a 2-fold reduction in the expression of Lyn, a Src-family member, while Cyclins B1 (6-fold) and B2 (6-fold) were significantly upregulated, which is consistent with progression of cells through the G2 and M-phases.

**Table 3:** Differential effect of GCs and TKIs on the expression of cell cycle regulators.

Genes	Control	6MP	PD16	PD17
**Checkpoint regulators**				
Wee1	1.0 ± 0.0	1.0 ± 0.5	0.9 ± 0.3	0.8 ± 0.3
Myt1	1.0 ± 0.1	2.7 ± 1.2*	0.9 ± 0.5	1.3 ± 1.0
Chk1	1.0 ± 0.1	4.2 ± 4.1	0.9 ± 0.3	1.3 ± 0.3
**Src family kinases**				
Lyn	1.0 ± 0.0	0.5 ± 0.2**	1.0 ± 0.4	0.8 ± 0.4
Fyn	1.0 ± 0.0	1.8 ± 1.2	1.1 ± 0.2	1.2 ± 0.3
**Cyclin dependent kinases**				
Cdk4	1.0 ± 0.1	2.2 ± 1.2	1.3 ± 0.5	1.2 ± 0.7
Cdk2	1.1 ± 0.1	1.9 ± 0.8	1.3 ± 0.7	1.3 ± 0.3
Cdk1	1.0 ± 0.0	5.1 ± 1.5**	1.1 ± 0.2	1.0 ± 0.3
**Cyclins**				
Cyclin D1	1.0 ± 0.1	0.8 ± 0.3	1.3 ± 0.5	1.3 ± 0.5
Cyclin E	1.0 ± 0.0	3.3 ± 1.4*	1.7 ± 0.8	1.5 ± 0.8
Cyclin B1	1.0 ± 0.0	5.6 ± 2.2**	1.1 ± 0.1	1.4 ± 0.7
Cyclin B2	1.0 ± 0.0	6.4 ± 2.4**	1.8 ± 0.2	1.6 ± 0.4

Purified rat beta cells were cultured for 6 days in absence or presence of the indicated compounds at 1μM. RNA was extracted on day 6 and used for qPCR analysis. Gene expression levels were normalized to 4 housekeeping genes, ΔΔCt values were calculated relative to the control condition. Data represent mean ± SD (n = 3–6). Statistical differences were analyzed by One-way ANOVA with Tukey’s correction for multiple comparisons

** p<0.01

* p<0.05 as compared to control.

### Discrepancy between stimulation of DNA-synthesis and increase in beta cell numbers: glucocorticoids versus checkpoint kinase inhibitors

Since we found no increase in beta cell numbers in TKI treated cells over 15 days, we decided to examine this in more detail. The dose-dependent effect of 6MP (0.01 nM to 1 μM) on DNA-synthesis and beta cell numbers was compared to the effect of PD16 and PD17 (0.1 and 1 μM). Cells were labeled with EdU between day 3 and 6 and the percent EdU-positive cells was determined on day 6. A change in cell number was evaluated after 15-days culture (**Table 4**). 6MP-exposed cells showed a dose-dependent recruitment into proliferation as demonstrated by a 2.4 to 5-fold increase in DNA-synthesis and a corresponding 1.1 to 1.5-fold increase in the number of living cells (**Table 4**).

**Table 4:** Effect of GCs and TKIs on EdU-incorporation, beta cell number and viability.

		Day 6		Day 15	
Compound	μM	% EdU+ cells	EdU+ cells (relative to control)	Living cell numbers (relative to control)	% Living cells
**Control**	−	4.0 ± 1.8	1.0 ± 0.0	1.0 ± 0.3	94 ± 4.7
**6MP**	0.00001	5.0 ± 3.3	1.2 ± 0.3	1.0 ± 0.1	95 ± 0.3
	0.0001	5.1 ± 3.4	1.3 ± 0.3	1.0 ± 0.1	94 ± 0.3
	0.001	8.9 ± 4.8***	2.2 ± 1.6**	1.1 ± 0.1*	93 ± 0.2
	0.01	14.8 ± 6.4***	3.8 ± 1.6***	1.3 ± 0.1**	94 ± 0.3
	0.1	19.5 ± 8.1***	4.9 ± 2.5***	1.5 ± 0.2***	94 ± 0.3
	1	19.8 ± 7.6***	5.0 ± 2.4***	1.5 ± 0.2***	88 ± 5.0
**PD173952**	1	12.8 ± 3.5***	3.2 ± 0.7***	1.0 ± 0.1	95 ± 1.8
**PD166285**	1	14.0 ± 5.8***	3.5 ± 0.8***	1.0 ± 0.1	93 ± 2.2

Rat beta cells were cultured without or with compounds at the indicated concentrations. EdU was added between day 3 and 6 and the percentage EdU positive cells was determined on day 6 and expressed relative to the control condition. Living cell numbers were determined on day 15 and expressed as a fold change relative to the number in the control condition. Data represent means ± SD (n = 9). Statistical differences between control and experimental conditions were analyzed by One-way ANOVA with Fisher LSD test

*** p<0.001

** p<0.01

* p<0.05 as compared to control.

**Fig 3:**
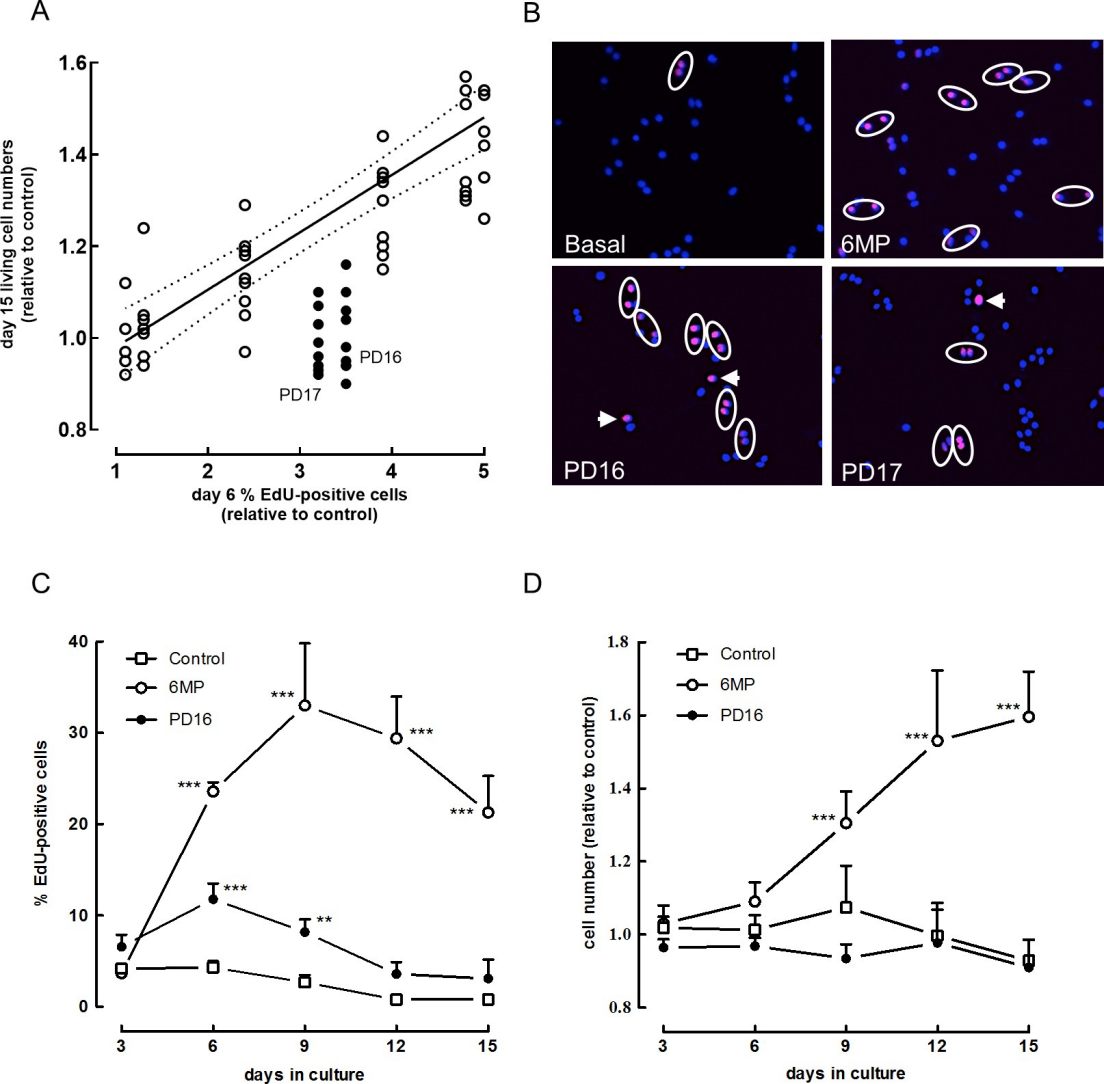
Correlation between EdU incorporation and beta cell numbers exposed to GC or TKIs. (A) Purified rat beta cells were cultured for 6 or 15 days with different concentrations of 6MP (0.1 nM-1μM), or the TKIs, PD16 or PD17, at 1μM. EdU was added on day 3 and measured on day 6. The absolute number of viable cells was determined on day 15. A positive correlation (r^2^ = 0.96, p<0.0001) was found between the dose-dependent change in percent EdU+ cells (relative to control cells, Table 4) and the corresponding increase in cell numbers measured on day 14 (Table 4) in 6MP treated cells (white circles, 0.1 nM to 1μM). The values for PD16 or PD17 are shown as black circles. Data represent the mean values of 9 independent experiments, with the 95% confidence interval. (B) Representative images showing EdU-labeling (magenta) and DAPI (blue). Purified rat beta cells were exposed for 6 days to 6MP, PD16 or PD17. An arrow denotes cells in S-phase, while a circle indicates couples of recently divided cells. (C, D). Purified rat beta cells were cultured without or with 6MP or PD16 at 1μM. EdU was added between day 1–3, 3–6, 6–9, 9–12 or 12–15. The percentage of EdU positive cells was calculated (C) and beta cell numbers relative to the control condition (D) were measured on day 3, 6, 9, 12 and 15. Data represent mean ± SD (n = 4). Statistical differences were analyzed by Two-way ANOVA with Tukey’s correction for multiple comparisons. * p<0.05, ** p<0.01, *** p<0.001, as compared to control.

Linear regression analysis showed a positive correlation (*r*^2^ = 0.96, p<0.001) between 6MP-induced EdU-incorporation measured on day 6 and the increase in cell numbers relative to control cells after 15 days (**Fig 3A**). This correlation implies that the percentage of EdU-positive cells measured after 6 days exposure to 6MP predicts the increase in cell numbers after 15 days. According to this correlation at least a 2-fold increase in the number of EdU-positive cells should be measured on day 6 in order to achieve a significant increase in cell numbers after 15 days (**Table 4**). The results shown in **Fig 3A** also point to a major discrepancy between the percentage of EdU-positive cells measured for PD16 or PD17, and their effect (or lack of effect) on cell numbers after 15 days. Despite an up to 3.5 fold (p<0.001) increase in the percent EdU-positive cells, cell numbers remained constant over 15 days (**Table 4**). The viability of the cells was not affected by the TKIs as measured by Hoechst/propidium iodide staining. Comparable observations were made at a concentration of 0.1 μM.

We further examined whether the discrepancy between increased DNA synthesis and the absence of an increase in cell numbers could be explained by a difference in viability of the newly formed daughter-cells. Cells were exposed to 6MP or TKI’s and labeled with EdU between day 3 and 6. As shown in **Fig 3B**, EdU-positive beta cells are mainly found as ‘couples’ after 72h labeling which suggests successful mitosis in all conditions. In parallel cultures, EdU was removed from the media on day 6 and the number of EdU-positive cells was determined 9 days later (thus on day 15) and compared to the number EdU-positive cells on day 6. For control cells, 87 ± 14% of the number of EdU-positive cells that were present on day 6 were found back 9 days later (day 15 of culture). For GC treated cells 80 ± 17% of the cells that were labeled with EdU on day 6 were still present 9 days later. For TKI-exposed cells these numbers 92 ± 19% for PD16 and 79 ± 13% for PD17, respectively. We conclude that viable EdU-labeled daughter cells that survive throughout culture are formed with both compound families.

We next performed a time-course analysis to compare the stimulatory effect of TKIs and 6MP on EdU-incorporation and beta cell numbers. Cells were incubated with the compounds and labeled with EdU between day 1–3, 3–6, 6–9, 9–12 or 12–15. EdU-positive and viable cell numbers were determined at the end of each labeling period. 6MP showed a pronounced stimulatory effect on DNA-synthesis from day 6 onward up to day 15, whereas the stimulatory effect of PD16 was highest between day 1 and 9 and leveled-off thereafter (**Fig 3C, Table 5**). Consequently a significant increase in cell numbers could be measured for 6MP from day 9 onward, but no change was observed for PD16 (**Fig 3D**). Comparable results were obtained with PD17 (**Table 5**). At none of these time points TKIs showed negative effects on the viability of the cells (**Table 5**).

**Table 5:** TKIs time dependently stimulate DNA-synthesis but fail to increase cell numbers.

	Days in Culture				
	3	6	9	12	15
**Control**					
% EdU+ cells	4.2 ± 0.3	4.3 ± 0.7	2.7 ± 0.8	0.8 ± 0.2	0.8 ± 0.3
Cell Number	2181 ± 259	2153 ± 285	2280 ± 346	2254 ± 120	1928 ± 321
FC Cell Number	1.0 ± 0.0	1.0 ± 0.0	1.1 ± 0.1	1.0 ± 0.1	0.9 ± 0.0
% Living cells	94 ± 2	98 ± 1	99 ± 0	98 ± 1	92 ± 3
**6MP**					
% EdU+ cells	3.7 ± 0.8	23.6 ± 6.3***	33.0 ± 6.8***	29.4 ± 4.6***	21.3 ± 4.0***
Cell Number	2257 ± 154	2290 ± 362	2732 ± 447	3457 ± 379***	3611 ± 132***
FC Cell Number	1.0 ± 0.0	1.0 ± 0.1	1.3 ± 0.1***	1.5 ± 0.1***	1.6 ± 0.1***
% Living cells	94 ± 3	97 ± 1	97 ± 0	94 ± 2**	93 ± 1
**PD16**					
% EdU+ cells	6.6 ± 1.3	11.8 ± 1.7***	8.2 ± 1.4**	3.6 ± 1.3	3.1 ± 2.1
Cell Number	2100 ± 185	2088 ± 236	2047 ± 203	2210 ± 131	2058 ± 115
FC Cell Number	0.9 ± 0.0	1.0 ± 0.0	0.9 ± 0.0**	1.0 ± 0.1	1.0 ± 0.1
% Living cells	94 ± 1	97 ± 1	96 ± 1	96 ± 1	94 ± 0
**PD17**					
% EdU+ cells	5.3 ± 0.2	11.2 ± 0.9***	6.9 ± 0.7*	2.7 ± 1.2	2.2 ± 0.7
Cell Number	2145 ± 211	2067 ± 220	2089 ± 248	2173 ± 170	2049 ± 74
FC Cell Number	1.0 ± 0.0	0.9 ± 0.0	1.0 ± 0.0*	1.0 ± 0.1	1.0 ± 0.0
% Living cells	94 ± 2	96 ± 1	96 ± 0	96 ± 1	95 ± 0

Purified rat beta cells were cultured without or with compounds at 1μM, EdU was added during day 1–3, 3–6, 6–9, 9–12 and 12–15. The numbers of living cells and EdU+ cells were counted at the indicated days and expressed as absolute cell number, fold change (FC) of cell numbers versus control or percent EdU+ cells, respectively. Data represent mean ± SD (n = 3). Statistical differences were analyzed by Two-way ANOVA with Tukey’s correction for multiple comparisons test

* p<0.05

** p<0.01

*** p<0.001, as compared to control.

Based on the percentages of EdU-positive cells measured throughout culture in combination with the fold increase of the number of cells compared to day 1, it can be calculated that in the presence of 6MP up to 75% of all beta cells that are initially present become responsive to the GCs and are recruited into proliferation, whereas maximally 10 to 15% of all cells respond to TKIs. In comparison, even in control cultures, in the absence of compounds, up to 7% of the beta cells enter proliferation. The stimulatory effect of the TKIs on beta cells (which is thus only a few percent above control) seems to be insufficient to generate a measurable increase of the number of beta cells over 15 days. We did not test longer culture periods.

As these results were obtained using FACS purified beta cells, we also examined the effect of 6MP on dispersed rat islet cell preparations (**Table 6**). The results confirm that it is the beta cells that proliferate and expand in response to glucocorticoids, whereas other cell types (mainly alpha cells) did not, but rather declined.

**Table 6:** Effect of glucocorticoids on dispersed rat islets cells.

	Days of culture		
	1	9	15
**Control**			
% Ins+	76 ± 2	85 ± 1	88 ± 9
FC Ins+ Cell Number	1.0 ± 0.1	1.1 ± 0.1	1.3 ± 0.1 ^#^
% Gluc+	23 ± 0	13 ± 1^#^	8 ± 1^##^
FC Gluc+ Cell Number	1.0 ± 0.1	0.6 ± 0.0^#^	0.4 ± 0.0^#^
**6MP**			
% Ins+	75 ± 6	85 ± 1	86 ± 10
FC Ins+ Cell Number	1.0 ± 0.1	1.2 + 0.0^#^	1.8 ± 0.4** ^##^
% Gluc+	23 ± 2	14 ± 1^#^	9 ± 1^##^
FC Gluc+ Cell Number	1.0 ± 0.1	0.7 ± 0.1	0.6 ± 0.0 ^#^

Dispersed rat islets cells were cultured without or with 6MP at 1μM. The number of insulin (Ins+) and glucagon (Gluc+) positive cells was measured after 1, 9 and 15 days of culture and expressed as a percentage of the total number of cells measured at the indicated days, or as fold change relative to the number of cells measured at day 1. Data represent mean ± SD (n = 3). Statistical differences were analyzed by Two way ANOVA with Tukey’s correction for multiple comparisons, or Fisher’s LSD test

* p<0.05

** p<0.01, 6MP as compared to control

^#^ p<0.05

^##^ p<0.01, as compared to day 1.

### Glucocorticoids prepare beta cells to pass the cell cycle restriction point

As shown in **Fig 3C**, 6MP failed to stimulate DNA-synthesis during the first three days (day 1–3). A rise in the number of EdU-positive cells was only observed after more than 3 days exposure. This suggests that GCs require a certain incubation time to prepare the beta cells to enter the cell cycle, which may be reflected in their effect on the expression of cell cycle regulators.

**Fig 4:**
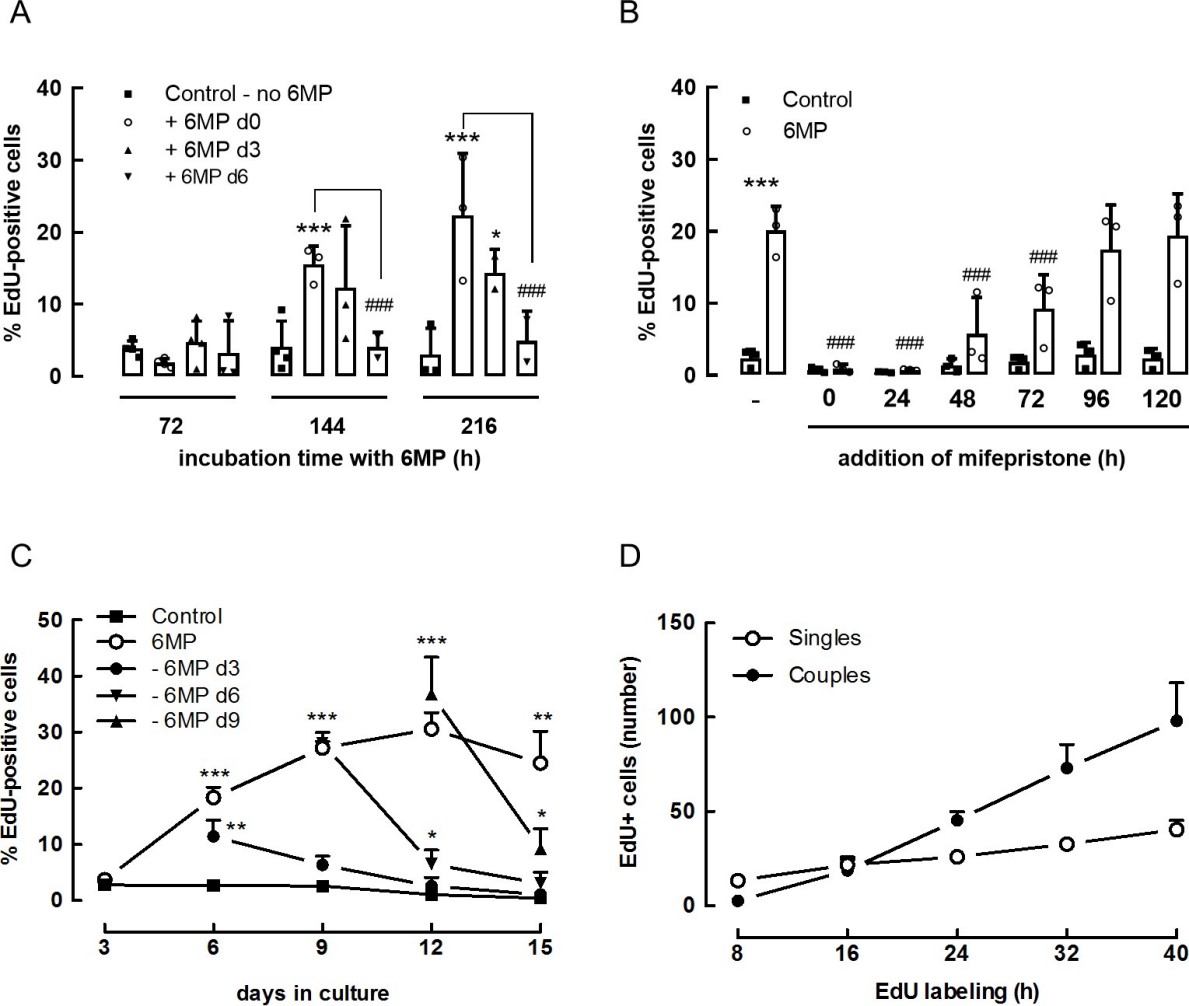
Effect of 6MP on DNA synthesis and cell cycle progression in rat beta cells. (A) Purified rat beta cells were cultured for up to 15 days. 6MP was administered at start of culture or after 3 or 6 days after seeding for an incubation period of 72, 144 or 216h. EdU was added during last 72h of culture after an exposure to 6MP of 72, 144 or 216h. Data represent mean ± SD (n = 3), the actual data points are also included (circles, squares, triangles, as explained in the figure). Statistical differences were analyzed by Two-way ANOVA with Tukey’s correction for multiple comparisons. * p<0.05, *** p<0.001 as compared to control. ^###^ p<0.001 as compared to the respective 6MP-control condition. (B) Purified rat beta cells were cultured 6 days without or with 6MP. Mifepristone was added immediately (0 h) or 24, 48, 72, 96 or 120h later. EdU was added between day 3–6 and percentage EdU positive cells were calculated on day 6. Data represent mean ± SD (n = 3), the actual data points are also shown (circles, squares, triangles). Statistical differences were analyzed by Two-way ANOVA with Tukey’s correction for multiple comparisons. *** p<0.001 as compared to control. ^###^ p<0.001 as compared to 6MP without mifepristone. (C) Purified rat beta cells were cultured for 15 days with or without 6MP. 6MP was washed away from the culture media after 3, 6, 9 or 12 days exposure. EdU was added for the proceeding 48 or 72h and percentage EdU positive cells were calculated on the indicated days. Data represent mean ± SD (n = 3). Statistical differences were analyzed by Two-way ANOVA with Tukey’s correction for multiple comparisons. * p<0.05, ** p<0.01, *** p<0.001, as compared to control. (D) Purified rat beta cells were cultured with 6MP and EdU was added on day 3. The absolute number of doublets and single EdU positive cells were determined 8, 16, 24, 32 and 40h after addition of the analogue. Data represent mean ± SD (n = 3).

We explored this idea further by delaying the administration of 6MP. 6MP was administered at start of culture (d0) or only after 3 or 6 days. EdU was added during the last 72h of culture after an exposure to 6MP of 72, 144 or 216h (**Fig 4A**).

When 6MP was added at start, or at day 3, more than 72h incubation with 6MP was required before an increase in EdU-positive cell numbers could be measured. In contrast, cells that received 6MP only 6-days after seeding showed no response at all. This observation suggests that the window of opportunity to induce proliferation with GCs is limited to the first 3 to 6 days of culture. The results also indicate that it takes at least 72h exposure before the first GC-responsive cells pass the G1/S checkpoint and enter the DNA synthesis phase.

To examine this in more detail we used mifepristone (1μM), a glucocorticoid receptor-antagonist, to block 6MP-induced signaling and to examine for how long beta cells need to be stimulated before they pass the G1 restriction point and become committed to the cell cycle. Cells were seeded and incubated with 6MP on day 0, and mifepristone was added immediately (0h) or 24, 48, 72, 96 or 120h later. EdU was added between day 3 and 6 as described before (**Fig 4B**). The results show that after 48h incubation with 6MP, addition of mifepristone, cannot any longer prevent an increase of the number of EdU-positive beta cells, which indicates that entry into DNA-synthesis in these cells does not any longer depend on sustained GC-signaling. Their number further increased with time and after 96h the number of EdU-positive cells detected after adding mifepristone was comparable to the number of EdU-positive cells without mifepristone. This indicates that most of the cells that enter the S-phase between day 3 and 6 (and thus become EdU-positive) passed the restriction point between 48 and 96h after adding 6MP. It can be concluded that minimally 48h exposure are required before the first GC-responsive beta cells are able to pass the restriction point.

A comparable conclusion was made from an alternative experiment in which 6MP was removed from the culture media after 3, 6, 9 or 12 days exposure (**Fig 4C**). When 6MP was washed out on day 3, a similar percentage of EdU-positive cells was measured on day 6 as in the continued presence of 6MP (11 ± 3% vs 18 ± 2%; p = 0.114), but the stimulatory effect was lost at later time points. Removal of 6MP at later time points (after 6, 9 or 12 days) did not affect the number of cells undergoing proliferation in the subsequent 72h. Thus, the percent cells that entered the S-phase without 6MP versus those with 6MP remained the same; day 9: 28 ± 2% vs. 27 ± 1%; day 12: 37 ± 7% vs. 31 ± 3%; day 15: 23 ± 3% vs. 25 ± 6%. In all cases, a decrease of more than 25% was measured during the subsequent 72h follow-up period (**Fig 4C**). From these results, we can conclude that GC-responsive cells pass the restriction point and thus continue to enter the DNA-synthesis phase even in the absence of 6MP over a period of about 48-72h. It also can be noted that from day 6 onwards, the percentage of cells that incorporates EdU after removal of 6MP remains higher than the basal control condition, which indicates a long lasting stimulatory effect.

As already shown in **Fig 2B** most EdU-positive beta cells are found as couples after 72h labeling with the thymidine analogue, with less than 14 percent as single cells (**S1 Table)**.

Single EdU-positive cells frequently showed an enlarged nucleus, indicating active DNA replication. This prompted us to evaluate the period of time needed between passing the G1/S checkpoint and completion of mitosis. Cells were incubated with 6MP on day 0 and EdU was added to the culture media between day 3–6. The number of EdU-positive single cells (often with an enlarged nucleus, characteristic for the S-phase of the cell cycle) and EdU-positive couples were counted manually 8, 16, 24, 32 and 40h after addition of EdU. The first couples EdU-positive cells were observed after 16h and their number further increased in the later time points resulting in almost double as many couples as single EdU-positive cells after 40h labeling (**Fig 4D**). This indicates that 16h are sufficient to go from G1/S to completion of mitosis. We did not test time points between 8 and 16h, so it cannot be excluded that the duration of the S-phase is still a bit shorter than 16h.

## Discussion

We previously described a high-content imaging method to document changes in absolute beta cell numbers during prolonged culture using serum free medium [8]. This method was used to identify compounds that trigger DNA-synthesis in rat and human beta cells and to examine the effect of such compounds on beta cell numbers during prolonged culture. In a recent report we described the identification of GCs as potent inducers of human and rat beta cell proliferation from a library with pharmacologically active compounds [9]. Incubation with 6MP, not only stimulated DNA-synthesis, but also led to a near doubling of functional beta cell numbers within 14-days that were able to restore insulin-secretion and glycemic control after transplantation in alloxan-induced diabetic mice. Moreover, starting from initially the same number of cells, double as much animals could be transplanted [9].

The work in this manuscript further emphasizes the importance of examining the effect of compounds that stimulate beta cell proliferation at the level of changes on cell numbers over a prolonged period. Although several classes of molecules and growth factors, such as small molecule glucokinase activators (GKAs), DPP-4 inhibitors, GLP-1 agonists, adenosine kinase inhibitors, GSK3β inhibitors, cAMP mediators, IGF-1 and prolactin, and others have been reported to stimulate beta cell proliferation [5–7,23,24], it remains to be seen whether these compounds actually lead to a measurable increase in cell numbers. At least some of these proliferation inducers were identified using BrdU, which might reflect DNA-damage and repair [25], rather than synthesis. GKAs for instance, have repeatedly been shown to stimulate beta cell replication, but remain unable to increase beta cell mass and only show a transient benefit in clinics [26–31]. Genetic overactivation of glucokinase, despite initially triggering replication, has been shown to cause DNA double-strand breaks and apoptosis [32]. Overexpression of HNF4alpha has been shown to initiate cell cycle entry, as is the case in our experiments with the checkpoint TKIs, but was unable to promote beta cell expansion [25]. None of these compounds were identified in our screening assay in which we only tested one concentration.

In the current manuscript we use GCs and checkpoint TKIs as an example to show the fraction of beta cells that needs to be recruited into DNA-synthesis and to undergo successful mitosis in order to be able to measure a significant increase of beta cell numbers in vitro within a relatively short time. These insights are important for approaches that aim to expand functional beta cells for replacement therapies.

By comparing the effect of GCs to TKIs, such as CHIR124, dasatinib, PD16 and PD17, that were as powerful as 6MP in stimulating DNA-synthesis, we found that checkpoint TKIs failed to increase beta cell numbers. This discrepancy could not be explained by a reduced overall viability of the cells, nor by a lack of viability of the newly formed daughter cells. Although the TKIs used in this study have been developed to override the cell-cycle checkpoints in cancer cells and to induce micronuclei formation, premature mitosis and apoptosis [33–37], we could not demonstrate any of these events in beta cells. On the contrary, our results show successful completion of mitosis with the formation and prolonged survival of newly-formed daughter cells.

A time-course analysis revealed that the selected TKIs stimulated DNA-synthesis only during the first 9 days of culture, but at later time points their stimulatory effect diminished. As a result, although both compound families seem to be equipotent in stimulating proliferation the first 6 days of culture, only 10% to 15% of all beta cells enter the S-phase over a period of 15 days when incubated with TKIs. This means that the maximal increase in cell numbers that can be expected with TKIs is only 15%, assuming that no cells die during culture. Our conclusion is that this fraction is insufficient to give a measurable increase of beta cell numbers in vitro within 15 days. We did not test longer incubation periods, because our results showed that TKIs do not recruit beta cells into a sustained proliferative activity, nor did they alter the mRNA levels of cell cycle regulators. Since we aim to identify compounds that increase the number of beta cells, we did not put further effort in demonstrating the effect of TKIs on cell cycle regulators at the protein level. Our observations seem to be in line with what has been observed in other cell types [33–37], namely that these TKIs act as cell cycle progression factors that allow a limited fraction of beta cells to bypass the G1/S checkpoint by changing the activation status of checkpoint kinases, such as Chk1, Myt1 or Src-kinases. In the same context, other screening efforts also identified cell cycle-dependent kinase inhibitors p18 and p21 as targets to promote cell cycle entry in human beta cell [38]. It remains to be examined whether these compounds would support beta cell expansion.

However, we also need to point out that our results were mainly obtained with rat beta cells. Given that the selected checkpoint TKIs also show a robust increase in DNA-synthesis in human beta cells (up to 7-fold above control), and the fact that the cyclins and cyclin dependent kinases that control beta cell proliferation may be different in rodents and humans [39], it remains to be examined whether checkpoint TKIs would be able to increase human beta cell numbers or could stimulate beta cell proliferation when administered in vivo. This is interesting since recent clinical trials have indicated potential benefits for diabetic patients [14,15]. In addition, TKIs, such as dasatinib and imatinib, have been reported to improve insulin sensitivity and glucose tolerance and to prevent the onset, or to lead to the remission of type 1 and type 2 diabetes in various animal models [40–45], attributed to their immunomodulatory activities and reduction of ER-stress [46,47]. A direct effect of these kinase inhibitors on human beta cell proliferation has not been reported before. Moreover, along the same line, inhibitors of Dual-Specificity Tyrosine Phosphorylation-Regulated Kinase 1A (DYRK1A), which also plays a role in repressing the cell cycle, have been identified as effective inducers of human beta cell proliferation and cell numbers [48–54]. Combined inhibition of DYRK1A, SMAD and Trithorax pathways was recently shown to induce replication in human cells [53]. It remains to be examined whether combination with checkpoint kinase inhibitors would also synergize to expand human and rat beta cells.

**Fig 5:**
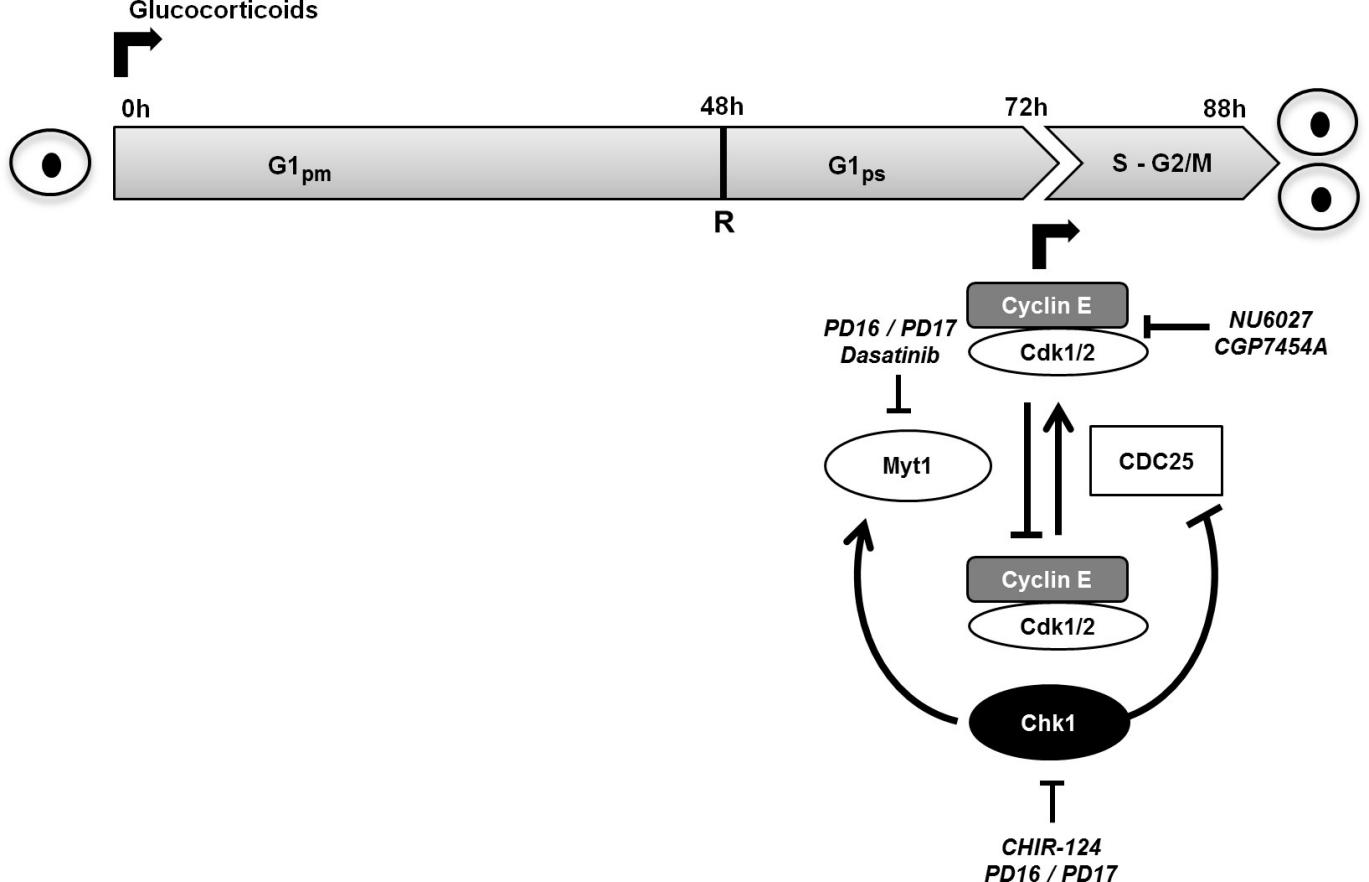
Schematic representation of key regulators involved in cell cycle progression of beta cells with plausible action of glucocorticoids and tyrosine kinase inhibitors. Glucocorticoids seem to act as proliferation competence-inducing factors that enable beta cells to cross the cell cycle restriction point (R) within 48h upon start of exposure. An additional 24h are needed to finish G1ps and to reach the G1/S-checkpoint which is controlled by Chk1 and Myt1 kinase which keep Cdk:cyclin E inactivated via an inhibitory phosphorylation. Inhibition of Chk1 or Myt1 or using specific inhibitors (CHIR-124, PD16, PD17 or Dasatinib) leads to the activation of the cdk1/2:cyclin E complex increases the number of cells involved in DNA-synthesis. Inhibition of Cdk1, and to a lesser extend Cdk2 inhibits DNA synthesis induced by 6MP and TKIs. After passing the G1/S checkpoint beta cells need a maximum of 16h to finish mitosis and to form two viable daughter cells. G1_pm_ = G1 post-mitotic phase; G1_ps_ = G1 pre-S phase.

Our finding that GCs directly stimulate beta cell expansion is quite controversial in view of the many negative effects on functional beta cell mass ascribed to these hormones [55–61], whereas some studies also point to an important role of glucocorticoid signaling in beta cell function and maturation [62,63]. Although prior studies reported beta cell proliferation upon *in vivo* GC-treatment, this was consistently explained as a side effect of steroid-induced insulin resistance and increased metabolic needs [64–66]. Our current results, summarized in **Fig 5** show, however, that these steroid hormones do not act as cell cycle progression factors, but rather as proliferation competence-inducing signals that prepare beta cells to pass the restriction point. In contrast to the examined checkpoint TKIs, GCs continue to recruit additional beta cells into proliferation throughout the whole culture period during which at least 50% and in some experiments even as much as 75% of all beta cells respond to 6MP. While many examples of induced cell cycle entry have been reported, we are not aware of molecules that showed similar efficiency as GCs to expand mature rat beta cells. In this study we therefore used 6MP to probe beta cell kinetics by taking advantage of the ability to time the administration of this mitogen. Our insights are summarized in **Fig 5**. An incubation period of minimally 48h seems required to enable the first cells to pass the restriction point after which cell cycle progression becomes independent of sustained glucocorticoid receptor signaling. Seventy-two hours are needed before the first increase in EdU-positive cells is observed. The duration of the G1ps phase (pre-S phase), which follows after passing the restriction point and in which cells prepare to pass the G1/S checkpoint thus takes approximately 24h. We also showed that it takes maximum 16h after entering the S-phase to complete mitosis and form two viable daughter cells. Therefore, we can estimate that it takes about 88h from the time of the addition of 6MP to the formation of daughter cells. This period is much longer than what has been deduced previously by Hija et al. from GKA-stimulated beta cells [67]. In these *in vivo* experiments 8h were required upon GKA stimulation to move from G0 to the restriction point and 5h to reach the S-phase. After entering the S-phase, another 14h were needed to complete mitosis [67], which is comparable to the 16h observed in our *in vitro* experiments.

Withdrawal of 6MP or addition of mifepristone, a glucocorticoid receptor antagonist, after 48-72h exposure had no effect on subsequent beta cell proliferation. This argues for the concept that GCs act as replication competence-inducing factors that prepare beta cells to proliferate and render them more receptive to cell cycle progression factors, such as glucose or GKA [9]. In this context it needs to be mentioned that our experiments were performed in serum free media supplemented with IBMX. Control experiments, however, confirmed that even in the absence of IBMX, which reduces cell viability and results in cell loss, GCs continue to trigger beta cell replication.

The observation that 6MP influences the expression of multiple G1/S-phase regulators further supports the idea that these steroid hormones prepare beta cells to acquire a proliferative phenotype. Cell cycle entry normally relies on inactivation of pRb by early G1pm (G1 post-mitotic phase) cdk:cyclin complexes such as Cdk4, Cdk6, D-cyclin, or on late G1ps (G1 pre-S-phase) cyclins and Cdks, such as Cdk1/2-cyclinA/E [68]. It were mainly the latter that were induced by 6MP. Initiation of the cell cycle and transition from G1 to S-phase in beta cells has been attributed to activation of Cdk4-cyclin D and Cdk2-cyclin E complexes and their interplay with Cdk inhibitors of the INK4/ARF and the CIP/KIP family [68]. Our data now suggest a central role for Cdk1 in G1/S-phase transition, as its chemical inhibition completely suppressed 6MP-induced beta cell expansion, whereas only partial inhibition was obtained using a Cdk2 inhibitor. The same observation was made in beta cells forced to undergo mitosis upon incubation with TKIs. A central role for Cdk1 in controlling G1/S-phase transition in rat beta cells is also in accordance with a recent report that showed that Cdk1:cyclin E were the most effective combination to induce cell cycle entry in human beta cells [69].

In conclusion, our results with GCs have important implications for the identification of drugs aimed at increasing functional beta cell mass. Such drugs must remain in the presence of beta cells for at least 48h to prepare the cells for proliferation. Since not all cells respond simultaneously, the administration has to be repeated or to be continued over a prolonged period in order to be able to expand a sufficient number of cells and the cells must remain responsive to the compounds The exact mechanism by which GCs stimulate beta cells to pass the restriction point remains to be established.

## Supporting information

S1 Fig Effect of 6MP and selected TKIs on DNA-synthesis beta cell cultures. Representative images taken with the Pathway Bioimager showing the effect of 6MP, PD16 and PD17 on DNA synthesis in FACS purified rat beta cell cultures. Purified rat beta cells were exposed for 6 days to the compounds and EdU was added the last 24 hrs. Immunostaining shows insulin (green), glucagon (red, positive cells indicated with an arrow) and EdU-labeling (magenta), nuclei were stained with DAPI (blue).
(TIF)

S1 Table Percentage of EdU+ cells found in couples or as single cells. Purified rat beta cells were cultured for 6 days in absence or presence of compounds at 1μM. EdU was added for 72h between day 3 and 6 and numbers of EdU-positive nuclei observed as doubles or as singles were determined on day 6. Statistical differences between control and experimental conditions were analyzed by oneway ANOVA with Fisher’s LSD test; * p<0.05, ** p<0.01, *** p<0.001. Data represent mean ± SD (n = 5).
(DOCX)
