# Supplementary figures and images for: Glucocorticoids and checkpoint tyrosine kinase inhibitors stimulate rat pancreatic beta cell proliferation differentially
Source: PLoS One. 2019 Feb 19;14(2):e0212210. doi: 10.1371/journal.pone.0212210 (PMC6380609; doi:10.1371/journal.pone.0212210)

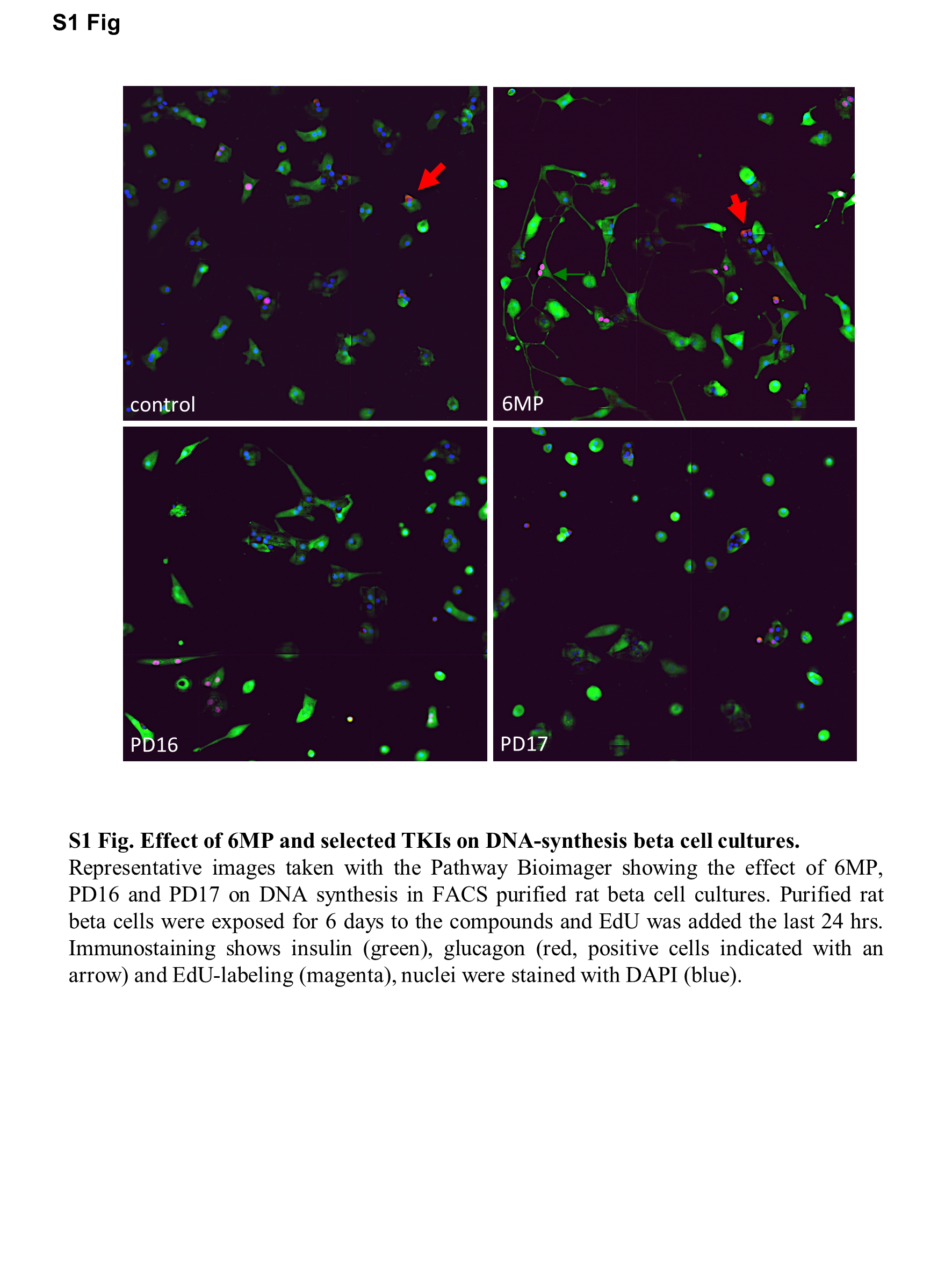

Supplement: S1 Fig — Representative images taken with the Pathway Bioimager showing the effect of 6MP, PD16 and PD17 on DNA synthesis in FACS purified rat beta cell cultures. Purified rat beta cells were exposed for 6 days to the compounds and EdU was added the last 24 hrs. Immunostaining shows insulin (green), glucagon (red, positive cells indicated with an arrow) and EdU-labeling (magenta), nuclei were stained with DAPI (blue). (TIF) [file pone.0212210.s001.tif]
