# Supplementary material for: Glucocorticoids and checkpoint tyrosine kinase inhibitors stimulate rat pancreatic beta cell proliferation differentially
Source: PLoS One. 2019 Feb 19;14(2):e0212210. doi: 10.1371/journal.pone.0212210 (PMC6380609; doi:10.1371/journal.pone.0212210)
Supplement: S1 Table — Purified rat beta cells were cultured for 6 days in absence or presence of compounds at 1μM. EdU was added for 72h between day 3 and 6 and numbers of EdU-positive nuclei observed as doubles or as singles were determined on day 6. Statistical differences between control and experimental conditions were analyzed by oneway ANOVA with Fisher’s LSD test; * p<0.05, ** p<0.01, *** p<0.001. Data represent mean ± SD (n = 5). (DOCX) [file pone.0212210.s002.docx]

**S1 Table. Percentage of EdU+ cells found in couples or as single cells.**

|  | **% EdU+ cells** | **% in Couples**  **mean ± SD** | **% in Singles**  **mean ± SD** |
| --- | --- | --- | --- |
| **Control**  **6MP**  **PD16**  **PD17** | 6 ± 1  33 ± 2***  10±2**  13±2** | 86 ± 2  90 ± 2 ***  88 ± 3 *  90 ± 1 ** | 14 ± 1  10 ± 2 ***  12 ± 3 *  10 ± 2 ** |

Purified rat beta cells were cultured for 6 days in absence or presence of compounds at 1µM. EdU was added for 72h between day 3 and 6 and numbers of EdU-positive nuclei observed as doubles or as singles were determined on day 6. Statistical differences between control and experimental conditions were analyzed by oneway ANOVA with Fisher’s LSD test; * p<0.05, ** p<0.01, *** p<0.001. Data represent mean ± SD (n = 5).
